# Repellent and Antifeedant Activities of Citral-Derived Lactones against the Peach Potato Aphid

**DOI:** 10.3390/ijms21218029

**Published:** 2020-10-28

**Authors:** Katarzyna Dancewicz, Antoni Szumny, Czesław Wawrzeńczyk, Beata Gabryś

**Affiliations:** 1 Department of Botany and Ecology, University of Zielona Góra, Szafrana 1, 65-516 Zielona Góra, Poland; b.gabrys@wnb.uz.zgora.pl; 2 Department of Chemistry, Wrocław University of Environmental and Life Sciences, Norwida 25, 50-375 Wrocław, Poland; antoni.szumny@upwr.edu.pl (A.S.); czeslaw.wawrzenczyk@upwr.edu.pl (C.W.)

**Keywords:** lactones, terpene derivatives, antifeedant, aphids, probing behavior

## Abstract

Citral is well known for its antimicrobial, antifungal, and insecticidal activities. Natural sesquiterpene α-methylenelactones also exhibit a broad spectrum of biological activities. The aim of the study was to explore the effect of structural changes to citral molecules on citral behavior-modifying activity towards *Myzus persicae*. Specifically, the effects of the introduction of a γ-lactone moiety and methylene groups in α and γ positions of the lactone ring were investigated. The lactones were obtained in five-step (saturated lactone and γ-methylenelactone) or six-step (α-methylenelactone and α,γ-dimethylenelactone) syntheses from citral. The synthetic procedures and physical and spectral data of the lactones are presented. The settling behavior of freely moving aphids in choice and no-choice situations was monitored. The probing behavior of tethered *M. persicae* using the Electrical Penetration Graph (EPG) technique was also analyzed. Citral appeared a strong repellent and pre-ingestive and ingestive probing deterrent to *M. persicae*. The incorporation of a lactone moiety caused the loss of the repellent activity. α-Methylenelactone inhibited aphid settling and probing activities at pre-ingestive and ingestive phases. The saturated γ-lactone and α,γ-dimethylenelactone were the settling post-ingestive deterrents to *M. persicae*, which did not affect aphid probing activity. γ-Methylenelactone did not affect aphid behavior.

## 1. Introduction

Plants infested by aphids may be affected directly, mainly because of fluid and nutrient removal, and indirectly by virus transmission. The Food and Agriculture Organization (FAO) estimates that between 20 and 40% of global crop yields are reduced each year due to the damage caused by plant pests and diseases [1]. The average yearly world crop loss due to aphids is estimated at least 2% of all losses on account of insect feeding [2]. Pre-harvest crop loss caused by aphids may reach over 50% in maize *Zea mays* L. and 80% in wheat *Triticum aestivum* L. (Poaceae), and 80% in *Brassica* sp. (Brassicaceae) [3]. The peach-potato aphid *Myzus persicae* (Sulz.) (Hemiptera: Aphididae) is one of the most noxious species. It can infest plants of over 40 different families including many economically important ones worldwide, and it is able to transmit over 100 plant viruses [4]. Considering various negative effects of conventional pesticide application, there is an increasing demand for more specific, indirectly-acting crop protection agents, such as insect behavior-modifying chemicals. Specifically, repellents, insect-growth regulators, oviposition inhibitors, and antifeedants have attracted a lot of attention as crop protection agents that, at least in part, might replace conventional insecticides in aphid control [5,6]. The most important discoveries included terpenoids of plant origin. Polygodial, applied in the field against *Rhopalosiphum padi* L., gave results comparable to the pyrethroid cypermethrin [5]. From a practical point of view, the use of plant-derived antifeedants on a large scale is not economic. Synthetic analogues of natural compounds are more accessible for application. 

Citral is a mixture of monoterpenoid aldehydes, predominantly geranial and neral, and is a component of essential oils of many plants. The highest content can be found in lemon myrtle *Backhousia citriodora* F.Muell., may chang *Litsea cubeba* (Lour.) Pers., or lemon tea-tree *Leptospermum liversidgei* R.T.Baker & H.G.Sm. [7,8,9]. Citral is well known for its antimicrobial, antifungal, and insecticidal activities [10,11,12,13]. Citral appeared toxic to the Mediterranean fruit fly *Ceratitis capitata* (Wiedemann), American cockroach *Periplaneta americana* (L.), and housefly *Musca domestica* L. [14,15,16] and repellent to termites *Coptotermes formosanus* Shiraki [17] and mosquitoes *Aedes aegypti* (L.) [18]. Citral also had a repellent effect against *M. persicae*, which was manifested in the significant decrease in time spent on leaves, decrease in total and mean time of penetration, and lower number of probes as compared to control; citral reduced also the total and mean probing time of aphids and their settling on the leaves [19].

The activity of behavior-modifying compounds is related to the presence of various functional groups. Often, natural antifeedants are lactones with one or more additional functional groups, including an α-methylenelactone moiety [20,21,22]. α-Methylenelactones are widespread in a nature, especially in the plants from Asteraceae and Euphorbiaceae families. They have also been found in bacteria algae, fungi, bryophytes, and lichens [23,24,25,26,27]. Natural sesquiterpene α-methylenelactones exhibits a broad spectrum of biological activities, namely cytotoxic, anticancer, and feeding-deterrent activities [28,29,30,31]. Our interest in the synthesis of α-methylenolactones was inspired by the insect antifeedant activity of the natural compounds.

The aim of the present study was to explore in detail the structure–activity relationships of citral and citral-derived α-methylene lactones at the plant–aphid interface. We focused on the behavioral responses of aphids, following the exogenous application of natural compound citral and its synthetic derivatives to the host plants. Specifically, we were interested in the effect of the introduction of a γ-lactone moiety into the citral structure, and methylene groups in the α and γ positions of the lactone ring. To reveal various aspects of citral and citral-derived lactone activities, we monitored the behavior of freely moving aphids in choice and no-choice situations. Additionally, we recorded the probing behavior of tethered aphids in a no-choice situation using the Electrical Penetration Graph (EPG) technique, which visualizes the movements of aphid mouthparts within individual plant tissues. The values of parameters derived from EPG recordings are reliable and accurate indicators of aphid behavioral responses to alteration in plant suitability due to exogenous application of xenobiotics.

## 2. Results

### 2.1. Synthesis of Citral-Derived Lactones

**Figure 1:**
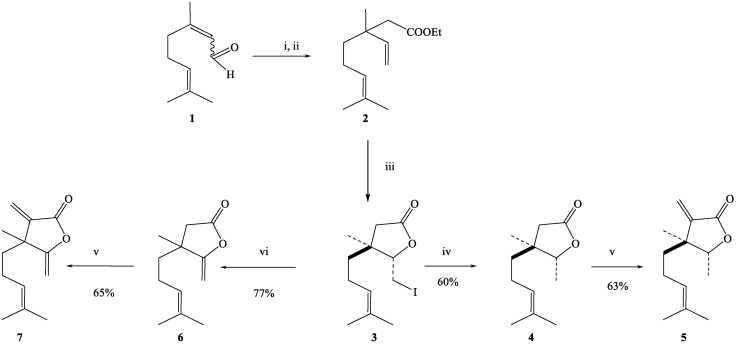
Synthesis of the studied lactones. (i) NaBH_4_ in MeOH; (ii) CH_3_C(OEt)_3_, EtCOOH, 138 °C; (iii) first KOH, MeOH then I_2_, NaHCO_3_ and isolation of trans-δ-iodo-γ-lactone on column chromatography; (iv) TBTH; (v) two-step synthesis a) MMC in DMF, b) N-methyl-N-phenylamine, 30% HCHO, CH_3_COOH, CH_3_COONa; (vi) DBU, CH_2_Cl_2_. Citral (**1**); γ,δ-unsaturated ethyl ester (**2**); δ-iodo-γ-lactone (**3**); β,γ-dimethyllactone (**4**); α-methylenelactone (**5**); γ-methylenelactone (**6**); α,γ-dimethylenelactone (**7**).

The lactones studied were obtained in five or six-step syntheses from citral (**1**, a mixture of geranial and neral 70 and 30% respectively), as shown in Figure 1.

The key compound in these syntheses, δ-iodo-γ-lactone (**3**), was obtained according to the halolactonization procedure of α,β-unsaturated isoprenoid ketones and allyl alcohols elaborated by our group [32,33,34,35]. In this procedure, citral was reduced with NaBH_4_ in methanol to a mixture of geraniol and nerol, which were subjected to Claisen–Johnson rearrangement with triethyl orthoacetate [36]. The γ,δ-unsaturated ethyl ester (**2**) was then hydrolyzed (KOH in methanol) to the corresponding known carboxylic acid [37], which was transformed in the iodolactonization process to the mixture of cis (15%) and trans (85%) δ-iodo-γ-lactones. Iodolactonization was carried out in basic conditions [38]. The mixture of iodolactones was then separated by column chromatography.

Only the pure trans-δ-iodo-γ-lactone (**3**) was isolated. β,γ-Dimethyllactone (**4**) was obtained by reductive dehalogenation of iodolactone (**3**) with tributyltin hydride [39]. α-Methylenelactone (**5**) was obtained from β,γ-dimethyllactone (**4**) in the carboxylation and decarboxylative methylenation process [40,41]. γ-Methylenelactone (**6**) was a product of dehydrohalogenation of iodolactone (**3**) with 1,8-diazabicyclo[5.4.0]undec-7-ene (DBU) [42]. α,γ-Dimethylenelactone (**7**) was synthesized from γ-methylenelactone (**6**) according to the same procedure as α-methylenelactone (**5**). All lactones were obtained as racemic mixtures. The structures of synthesized compounds were confirmed by their spectral (^1^H NMR, IR and EI-MS) data. The physical and spectral data of the lactones **3**–**7** as well as the detailed procedures of their syntheses are presented in Supplementary Material.

### 2.2. Repellent and Feeding Deterrent Activities against Myzus Persicae

#### 2.2.1. Aphid Settling (Choice-Test for Freely Moving Aphids)

**Figure 2:**
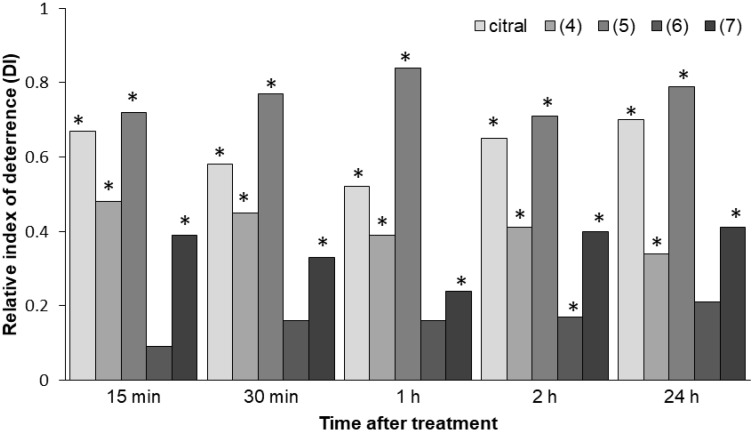
Relative indices of deterrence (DI) of citral and citral-derived lactones towards *Myzus persicae*; β,γ-dimethyllactone (**4**), α-methylenelactone (**5**), γ-methylenelactone (**6**), α,γ-dimethylenelactone (**7**). Asterisk denotes statistically significant differences at *p* ≤ 0.05 (Student *t*-test); DI = (C − T)/(C + T); DI < 0: attractant activity; DI > 0: deterrent activity.

The aphid settling bioassay allows us to study aphid host preferences under semi-natural conditions. Aphids settle on a plant only when they accept it as a food source [43]. Therefore, the number of aphids that settle on a given substrate is a good indicator of its suitability. In this experiment, aphid settling responses to citral (**1**), β,γ-dimethyllactone (**4**), α-methylenelactone (**5**), γ-methylenelactone (**6**), and α,γ-dimethylenelactone (**7**) were studied. Compounds γ,δ-unsaturated ethyl ester (**2**) and δ-iodo-γ-lactone (**3**) were the intermediate products in the synthesis of methyl- and methylenelactones and were not the subject of the present study. Significantly fewer aphids settled on the leaf halves treated with citral, β,γ-dimethyllactone (**4**), α-methylenelactone (**5**), and α,γ-methylenelactone (**7**) than on control halves (Table 1). This behavior did not change over the course of time; the freely moving aphids avoided the related sections of the leaves soon after the onset (15 min after aphids had access to the leaves) and until the end of the experiment (24 h after aphids had access to the leaves). The indices of deterrence (DIs) were the highest on citral and α-methylenelactone (**5**)-treated leaves and reached 0.7 and 0.8, respectively, 24 h after aphids had access to the leaves. In contrast, when γ-methylenelactone (**6**) was applied, aphids did not show preferences for either control or treated substrate during the twenty-four-hour period (Table 1, Figure 2).

#### 2.2.2. Aphid Early Behavioral Responses (No-Choice Test for Freely Moving Aphids)

In this experiment, the effects of citral, β,γ-dimethyllactone (**4**), α-methylenelactone (**5**), and α,γ-dimethylenelactone (**7**) on aphid behavior during the initial 15 min of contact with the treated substrate were studied. These compounds showed activity in the settling choice test for freely moving aphids. Compounds γ,δ-unsaturated ethyl ester (**2**) and δ-iodo-γ-lactone (**3**) were the intermediate products in the synthesis of methyl- and methylenelactones and were not the subject of the present study.

*M. persicae* started to probe within 11.6–20.9 s from the commencement of the experiment, irrespective of the treatment (Table 2). The total time on the leaf, the total probing time, and the mean duration of probes were significantly shorter on citral-treated leaves than on control and α,γ-dimethylenelactone (**7**)-treated leaves, and the total time on the leaf and the total probing time were significantly shorter on citral-treated leaves than on β,γ-dimethyllactone (**4**)-treated leaves. The number of probes was reduced on leaves treated with α,γ-dimethylenelactone (**7**) in comparison to control. On leaves treated with α-methylenelactone (**5**), the parameters related to aphid behavior showed intermediate values in relation to aphids on control, citral, β,γ-dimethyllactone-, and α,γ-dimethylenelactone-treated leaves (Table 2).

#### 2.2.3. Aphid Probing and Feeding (No-Choice Test for Tethered Aphids)

In this experiment, the effects of citral (**1**), β,γ-dimethyllactone (**4**), and α-methylenelactone (**5**), and α,γ-methylenelactone (**7**) on aphid behavior were studied. These compounds showed activity in the settling choice test for freely moving aphids. Compounds γ,δ-unsaturated ethyl ester (**2**) and δ-iodo-γ-lactone (**3**) were the intermediate products in the synthesis of methyl- and methylenelactones and were not the subject of the present study. The 8-h EPG monitoring demonstrated various phases of aphid probing behavior in plant tissues: non-probing, when aphid stylets were outside plant tissues, pathway phase “C” that represents probing within peripheral plant tissues, epidermis and mesophyll, and phloem phase “E” that indicated stylet position in sieve elements. The “F” (“derailed” stylet movements) and “G” (xylem uptake) waveform patterns occurred only incidentally. Due to rare occurrence in all experimental situations, the periods of “F” were included in pathway phase “C” in all calculations. Phloem phase “E” was composed of “E1” and “E2” waveform patterns and represented watery salivation and sap ingestion, respectively.

**Figure 3:**
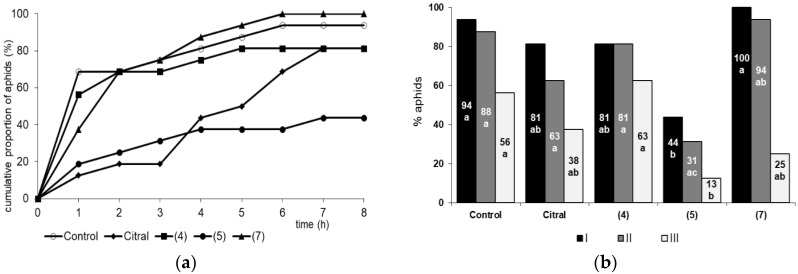
*Myzus persicae* success rate in finding phloem sieve elements and feeding on phloem sap on plant leaves treated with citral and citral-derived lactones: (**a**) Cumulative proportion of aphids that reached sieve elements during the 8-h access to plants. (**b**) Proportion of aphids that reached sieve elements (I), showed sustained sap ingestion E2 >10 min (II), and showed sustained sap ingestion during the first probe (III); β,γ-dimethyllactone (**4**), α-methylenelactone (**5**), α,γ-dimethylenelactone (**7**); *n* = 16; different letters show significant differences among aphids on plant leaves treated with citral and citral-derived lactones at *p* < 0.05 (Kruskal–Wallis test).

**Figure 4:**
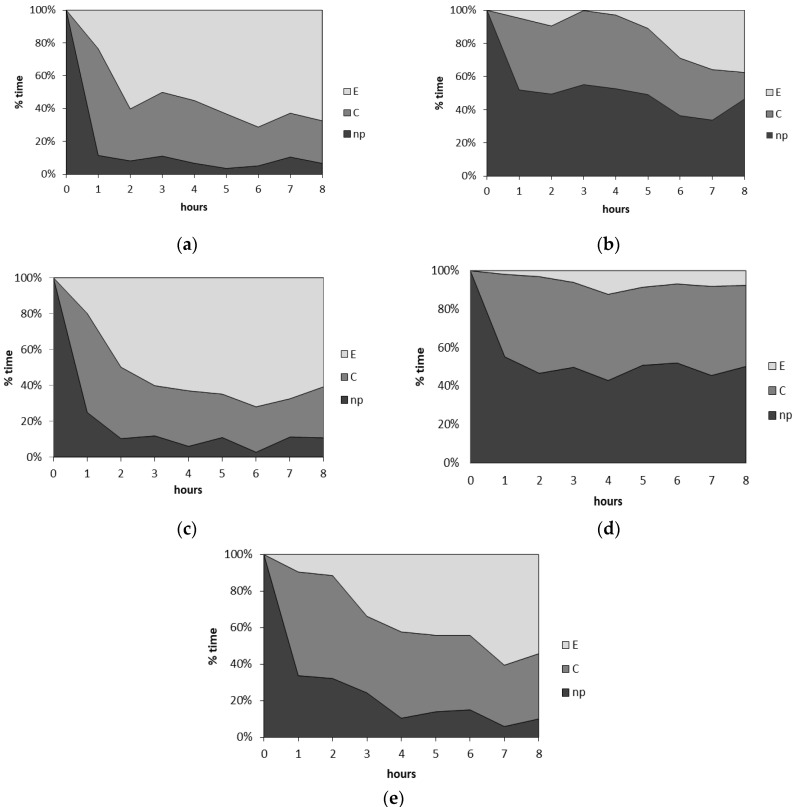
Sequential changes in *Myzus persicae* probing activity on *Brassica rapa* subsp. *pekinensis* on (**a**) control plants and plants treated with (**b**) citral, (**c**) β,γ-dimethyllactone (**4**), (**d**) α-methylenelactone (**5**), and (**e**) α,γ-dimethylenelactone (**7**) during the 8 h EPG monitoring of aphid probing. np—non-probing, C—aphid stylet activities in non-phloem tissues, E—aphid stylet activities in phloem tissues.

The typical behavior of *M. persicae* on the control untreated plants consisted predominantly of active probing (93% of the experimental time), which comprised mainly activities associated with phloem phase (62% of the probing time; phloem phase index = 0.62) (Table 3). Probing was rarely interrupted: the 7.4 h of probing was divided into 11.5 probes on average. The first probe was usually 1.7 h long. The sieve elements were reached by 70% of aphids within the first hour after having access to plants (Figure 3a), and the average for all aphids was 1.5 h after the onset of probing (Table 3). Nearly all (94%) aphids reached sieve elements, and nearly all aphids (88%) showed long (ca. ≤ 3-h) periods of sustained waveform E2, indicating successful feeding on untreated plants. Fifty-six percent of aphids showed sustained feeding during the first phloem phase (Figure 3b). The first sustained sap ingestion phase was nearly four hours long. The contribution of watery salivation to the phloem phase was 1.3% (phloem salivation index = 0.01) (Table 3). The proportion of phloem phase increased gradually in the course of time to reach 70% of all aphid activities at the end of the experiment (Figure 4a).

On plants treated with citral, the total duration of probing, the total duration of phloem phase, and the duration of the first sustained sap ingestion phase were significantly lower than in control (1.8, 3.5, and 2.4 times lower, respectively) (Table 3). The time from the first probe to the first phloem phase, the total number of probes, the number of probes, and the duration of no probing before the first phloem phase were higher than those of the control (2.5, 3.4, 4.7, and 8.4 times higher, respectively) (Table 3). Nearly 20% of aphids failed to locate sieve elements, and 40% failed to sustain the feeding on phloem sap (Figure 3a,b). Non-probing was the main aphid activity throughout the first four hours of the 8 h experiment (Figure 4b).

On β,γ-dimethyllactone (**4**) and α,γ-methylenelactone (**7**)-treated leaves, aphid behavior was similar to the control (Table 3, Figure 3a,b and Figure 4c,e). The contribution of probing in non-phloem and phloem tissues was comparable to control throughout the duration for the 8 h experiment (Figure 4c,e).

On plants treated with α-methylenelactone (**5**), the most distinct modification of aphid behavior during probing (EPG experiments) was observed (Table 3, Figure 3a,b and Figure 4d). In comparison to control plants, the total time of probing was significantly shorter (50% of 8 h experimental time, while on control plants—93%), aphids penetrated mainly peripheral tissues (80% of probing time, control—35%), and the total duration of phloem phase occupied 12% of the probing time (Table 3). The time before reaching phloem vessels was 1.7 times longer than that in the control, and 44% of that time was spent in non-penetration (Table 3). Probes preceding the first phloem phase on α-methylenelactone (**5**)-treated plants were numerous (2.5 as many as on control plants) but short, usually not longer than 2 or 10 min (57% of all probes). Moreover, 56% of aphids failed to reach phloem vessels; only 10% of aphids reached the phloem phase during the first hour after access to the plants (Figure 3a). Among those that got through to the phloem there were ones that did not succeed in ingesting sap for longer than 10 min (no sustained sap ingestion in over 30% of aphids that reached sieve elements) (Figure 3b). The phloem phase was marginal in aphid probing throughout the 8 h experiment (Figure 4d). The high proportion of salivation during penetration of phloem vessels (19% of all activities in sieve elements; phloem salivation index = 0.19) was noteworthy and significantly almost 20 times higher than that of the control (Table 3).

## 3. Discussion

In the present study, we discovered that citral and citral-derived lactones evoked different behavioral responses of *M. persicae*. Citral appeared a strong repellent and probing deterrent to *M. persicae*. The free aphids refused to probe and settle on citral-treated leaves. In addition, the tethered aphids were reluctant to probe, although they had a limited possibility to move away from the treated leaf. When they did probe, the stylet activities were restricted mainly to non-phloem tissues, and the probes were relatively short. Although with considerable delay, *M. persicae* individuals were generally able to reach phloem vessels on citral-treated leaves. However, the duration of sap ingestion was relatively low. β,γ-Dimethyllactone (**4**) appeared a settling deterrent to *M. persicae*. The free aphids refused to settle on the β,γ-dimethyllactone (**4**)-treated leaves, but the probing activity was not restrained. *M. persicae* spent similar time on probing activities in non-phloem tissues, reaching sieve elements in similar time, and consumed similar amount of sap as on control untreated plants. α-Methylenelactone (**5**) acted as a settling, probing and feeding deterrent. On α-methylenelactone (**5**)-treated leaves, the free *M. persicae* did not settle, and the probing activity of the tethered individuals was significantly limited. The tethered aphids were unwilling to probe, and when they did insert the stylets into plant tissues, the probes were relatively short and usually terminated before the stylets reached sieve elements. In consequence, many aphids failed to reach the food source. At the same time, many of those aphids that did reach sieve elements, did not accept the phloem sap readily. γ-Methylenelactone (**6**) did not affect the behavior of the free as well as the tethered *M. persicae*. α,γ-Dimethylenelactone (**7**) can be considered a settling deterrent. The tethered aphids did not avoid probing in the treated plants, and the feeding success was relatively high. Nevertheless, despite the unrestrained probing and feeding activities, many *M. persicae* individuals did not settle on the treated plants.

Insect behavior-modifying chemicals, such as repellents and antifeedants, change the host plant selection behavior, which may result in the reduction of crop losses caused by these herbivores [44,45,46]. Repellents are volatile chemical signals that elicit a negative displacement in the recipient [47], while antifeedants or feeding deterrents (phagodeterrents) are pre-ingestive feeding inhibitors that act through gustatory receptors and evoke rejection of the plant material by changing the sensory code from “acceptable” to “unacceptable” [48]. Both the repellents and antifeedants are essential components of “push-pull” strategies in Integrated Pest Management programs [46,49]. The feeding deterrents may modify the aphid host plant selection and acceptance processes by (i) interacting with taste receptors before the mouthparts stylets reach the plant sieve elements, (ii) affecting the phloem sap ingestion activity, and (iii) restraining physiological processes following the consumption of the sap [48]. These levels of antifeedant activities are defined as “pre-ingestive-” “ingestive”, and “post-ingestive” inhibition, respectively. Mechanisms of deterrence may act separately or in combination, and the results depend on insect species [48], which was the case in the present study. Citral was the only of the studied compounds that apparently had repellent properties, which was manifested in *M. persicae* movements away from the treated leaves. Citral also showed the pre-ingestive and ingestive deterrent activities, which were expressed in the avoidance of probing in non-phloem tissues and feeding on phloem sap, respectively. The structural alterations of citral molecules that led to various methylenelactones caused the loss of the repellent activity. None of the citral-derived lactones discouraged the aphids from the initiation of probing on the treated leaves. At the same time, different modifications had different impact on the compound deterrent activity. The strongest effect of lactonization and incorporation of α-methylene group on the compound activity was observed in α-methylenelactone (**5**). α-Methylenelactone (**5**) inhibited the aphid foraging activity at both pre-ingestive and ingestive phases of probing. The high proportion of salivation during the phloem phase in aphids on α-methylenelactone (**5**)-treated leaves suggests not only the deterrent but also probably toxic properties of α-methylenelactone (**5**). The enzymes present in the saliva may aid in metabolizing toxic or deterrent allelochemicals encountered during sap ingestion [50]. However, this hypothesis needs confirmation in further studies involving dose-dependent aphid responses. Under natural conditions, the high proportion of salivation during the consumption of the phloem sap is typical for aphid behavior on unsuitable or non-host plants [51,52,53]. Similar effects on aphid behavior during the phloem phase were revealed when aphids were offered plants treated with natural and synthetic compounds, e.g., piperitone-derived lactones, *cis*-jasmone and its derivatives, β-thujone and its derivatives, or farnesol and nerolidol [32,54,55,56]. The settling deterrent activities of β,γ-dimethyllactone (**4**) and α,γ-dimethylenelactone (**7**) were probably due to the post-ingestive inhibition. Neither the probing nor the feeding of *M. persicae* was disturbed by the application of β,γ-dimethyllactone (**4**) and α,γ-dimethylenelactone (**7**), which means that aphids consumed a considerable amount of sap during the experimental period. The rate of ingestion of the phloem sap is constant; therefore, the amount of the consumed sap depends on the duration of ingestion [57]. Sap consumption by *M. persicae* on β,γ-dimethyllactone (**4**)- and α,γ-dimethylenelactone (**7**)-treated plants was the major activity from the third hour after aphids had access to the plants until the end of the experiment. The avoidance of the treated leaves during settling might have been the delayed effect of consuming the toxic sap from β,γ-dimethyllactone (**4**)- and α,γ-dimethylenelactone (**7**)-treated leaves. This explanation, though, needs further study.

In conclusion, our study demonstrated that citral and certain citral-derived methyl- and methylenelactones act as repellents and feeding deterrents towards *M. persicae*, depending on their molecular structure. The natural compound citral exhibited the broadest aphid behavior-modifying activity and acted as a repellent and a pre-ingestive and ingestive deterrent. α-Methylenelactone (**5**) acted as a pre-ingestive and ingestive deterrent, while β,γ-dimethyllactone (**4**) and α,γ-dimethylenelactone (**7**) acted as post-ingestive deterrents. After having contact with citral or the citral-derived lactones—β,γ-dimethyllactone (**4**), α-methylenelactone (**5**), and α,γ-dimethylenelactone (**7**)—before or during probing in plant tissues, *M. persicae* walked away and did not settle on the treated leaves. Taking into account the described aphid behavior-modifying activities of citral and citral-derived lactones, essentially citral and the citral-derived α-methylenelactone (**5**) may be recommended for prospective practical application in aphid control, especially in “push-pull” strategies as the “push” elements applied individually or in combination. By limiting aphid activities prior to and/or during early stages of probing in peripheral plant tissues, citral and α-methylenelactone (**5**) may reduce plant infestation and limit the transmission of non-persistent and persistent viruses. Nevertheless, a detailed investigation on the concentration and persistence of the studied molecules in plant tissues as well as the dose–effect and synergy relationships should be established in an independent supplementary application-oriented study.

## 4. Materials and Methods

### 4.1. Synthesis of the Lactones

#### 4.1.1. Reagents

All reagents used were purchased from Sigma-Aldrich (Poznań, Poland) and citral from UQF Sp. z o.o. (Wrocław, Poland). Used solvents (analytical grade) were purchased from Archem (Wrocław, Poland).

#### 4.1.2. Purification and Analysis

Products were purified by preparative column chromatography on silica-gel (Kieselgel 60, 230–400 Mesh Merck) (Darmstadt, Germany) with a mixture of hexane and diethyl ether (in various ratios) as eluents. The progress of reaction and purity of products were checked by TLC and gas chromatography. Analytical TLC was performed on Merck Kieselgel plates with a mixture of hexane and diethyl ether as eluents. Compounds were visualized by dipping the plates in solution of 1% Ce(SO_4_)_2_ and 2% H_3_[P(Mo_3_O_10_)_4_] in 10% H_2_SO_4_. Finally, plates were heated with a hair-dryer. Gas chromatography was carried out on Varian-Chrompak 3380 (FID detector with helium as a carrier gas) on a HP-1 (30 m × 0.25 m × 0.25 μm film) column.

Refractive indices of products were measured on an Abbe refractometer (Carl Zeiss, Jena, Germany).

#### 4.1.3. Spectroscopic Analyses

Nuclear magnetic resonance (NMR) spectra were recorded in CDCl_3_ or in C_6_D_6_ solutions on a Bruker Avance II 300 MHz Bruker spectrometer (Bruker, Rheinstetten, Germany) with signals of residual solvents as references for chemical shifts.

IR spectra were recorded for liquid films on a Mattson IR 300 Thermo-Nicolet spectrophotometer (Waltham, MA, USA).

EI-MS spectrum: Gas chromatography of isolated compounds was performed on Varian CP-3800/Saturn 2000 apparatus (Varian, Walnut Creek, CA, USA) equipped with a Zebron ZB-5 MSI (30 m × 0.25 mm × 0.25 µm) column (Phenomenex, Shim-Pol, Poland). The GC oven temperature was programmed from 100 °C to 200 °C at rate 10.0 °C, then to 290 °C at rate 15.0 °C, and held for 5 min. Scanning was performed from 35 to 550 m/z in electronic impact (EI) mode at 70 eV. Samples were injected at a 1:10 split ratio, and helium gas was used as the carrier gas at a flow rate of 1.0 mL·min^−1^. Spectra were recorded in electron impact mode. 

### 4.2. Biological Studies

#### 4.2.1. Cultures of Aphids and Plants

Laboratory culture of peach-potato aphid *Myzus persicae* (Sulz.) was maintained on Chinese cabbage *Brassica rapa* L. ssp. *pekinensis* L. in laboratory at 20 °C, 65% r.h., and L16:8D photoperiod. One- to seven-day-old apterous females of *M. persicae* and three-week-old plants with 4–5 fully developed leaves were used for experiments. All experiments were carried out under the same conditions of temperature, relative humidity, and photoperiod as described for aphid and plant cultures. The bioassays were started at 10–11 a.m.

#### 4.2.2. Preparation and Application of Compounds

To mimic the natural environment under laboratory conditions, citral and citral-derived α-methylenelactones were offered to aphids by application through their host plants. Preparation and application of the compounds followed the procedure described by Polonsky et al. [58] and Powell et al. [59]. Briefly, each compound was dissolved in 70% ethanol to obtain a 0.1% solution. For all compounds, 0.01 mL/cm^2^ was applied on the adaxial leaf surface with a fine brush. Control surfaces were treated with 70% ethanol that was used as a solvent for the studied compounds. There is no effect of ethanol application on aphid probing behavior and plant condition [60]. All biological assays were performed 1 h after the application of the compounds to allow the solvent to evaporate.

#### 4.2.3. Aphid Settling Deterrent Activity (Choice-Test for Freely Moving Aphids)

This bioassay allows a study of aphid host preferences under semi-natural conditions. Aphids settle on a plant only when they accept it as a food source [43]. Therefore, the number of aphids that settle on a given substrate is a good indicator of its suitability. The settling behavior of *M. persicae* was studied using a procedure of the “half-leaf test” [58]: the compounds were applied on one half of the leaf; the other side of the midrib was coated with 70% ethanol and acted as a control. Aphids (8 replicates, 20 viviparous apterous females/replicate) were placed on the main nerve of a leaf, which allowed a choice between equal areas of treated and control surfaces. Aphids that settled on each side of the midrib, i.e., aphids that did not move and had their antennae directed backwards, which indicated probing [61], were counted at 15 min, 30 min, 1 h, 2 h, and 24 h intervals after access to the leaf. Aphids that settled at the midrib or were on the dish were not counted. The relative index of deterrence (ID) was calculated [60]: ID = (C − T)/(C + T), where C represents the number of aphids settled on the control half of the leaf, and T is the number of aphids settled on half of the leaf coated with the tested compound. The ID values may range from “−1” (very good attractant) to “1” (very good deterrent).

#### 4.2.4. Aphid Repellent Activity (No-Choice Test for Freely Moving Aphids)

Aphid repellent activity was studied by direct observation of the freely moving aphids on a leaf treated with the studied compounds, using the SONY SSC-DC50AP video camera (Tokyo, Japan), OLYMPUS SZ-CTV microscope, and OLYMPUS—DP-Soft v. 3.1 software (Tokyo, Japan). The experiment was carried out for 15 min (16 aphids/compound and control). Only compounds that significantly affected aphid settling in the choice-test were studied. The time spent on the leaf and the duration of probing were recorded based on the relationship between antennal and body movements and penetration of the stylets as described by Hardie et al. [61]. The position of antennae parallel to the abdomen and the cessation of body movements were associated with stylet penetration. The total time spent by aphids on leaves, total probing time, number of probes, mean probing time, and time from the beginning of experiment to the first probe were determined from this experiment. These parameters reflect the suitability of a plant as a host for the aphids. In the case of the artificially applied chemicals to the surface of the leaf, the shorter time that aphids spent on the leaf during the initial 15 min in comparison to the control may indicate the repellent properties of the given compound.

#### 4.2.5. Probing and Feeding Deterrent Activity (No-Choice Test for Tethered Aphids)

The probing and feeding deterrent activity of citral and citral-derived lactones to *M. persicae* was monitored using the Electrical Penetration Graph (EPG) technique, which provides a unique opportunity to reveal aphid mouthpart stylet activities in plant tissues [58,62,63]. The parameters describing aphid behavior during probing and feeding, such as time spent on probing activities, the occurrence and timespan of phloem sap uptake phases, the number of probes, etc., perfectly describe plant suitability [62]. Any difference in the values of these parameters in relation to the most favorable control results exposes the activity of chemical or physical mechanisms in individual plant tissues [64,65,66,67]. The EPG experimental set-up consists of an aphid and a plant that are included in an electric circuit, which is completed when the aphid inserts its stylets into the plant. Weak voltage is supplied in the circuit, and all changing electric properties are recorded as EPG waveforms. The waveforms have been correlated with individual aphid probing behaviors and stylet position in plant tissues [62,63]. 

In the present study, one- to seven-day-old adult apterous females of *M. persicae* and three-week-old plants with four to five fully developed leaves were used for all experiments according to the standard procedure applied in similar studies [32,53,55,65,66]. Aphids were connected to a golden wire electrode with conductive silver paint and starved for 1 h prior to the experiment. Probing behavior of apterous *M. persicae* was monitored for 8 h continuously with a Giga-8 DC EPG with 1 GΩ of input resistance (EPG Systems, Wageningen, The Netherlands) and Stylet+ software (www.epgsystems.eu). Each aphid was given access to a freshly prepared leaf of an unused plant, which means that each plant and each aphid were used only once. One aphid–plant combination was considered a replication. Giga-8 DC EPG allows the recording of 8 samples simultaneously. Two Giga-8 DC EPG systems were used to obtain 16 replications for each tested substance and control, daily. Considering the aphid diurnal activity, the EPG experiments commenced at 10–11 a.m. MEST (Middle European Summer Time). Only compounds that significantly affected aphid settling in the choice-test were studied, which were citral (**1**), β,γ-dimethyllactone (**4**), α-methylenelactone (**5**), and α,γ-dimethylenelactone (**7**). Compounds γ,δ-unsaturated ethyl ester (**2**) and δ-iodo-γ-lactone (**3**) were the intermediate products in the synthesis of methyl- and methylenelactones and were not the subject of the present study. All experiments were carried out under the same conditions of temperature, relative humidity, and photoperiod as described for the rearing of plants and aphids, which were at 20 °C, 65% r.h., and L16:8D photoperiod.

The following aphid behaviors related to mouthpart position in or out of the plant tissues were distinguished: non-probing, which represents aphid stylets outside the plant tissues; pathway phase “C”, which represents the movement of aphid stylets within epidermis and mesophyll; phase “F”, which represents unidentified (“derailed”) stylet movements within apoplast; xylem phase “G”, which represents active xylem sap uptake; a phloem phase consisting of watery salivation “E1” and passive ingestion of phloem sap “E2”. “F” occurred sporadically irrespective of treatment; therefore, these activities were analyzed together with phase “C” and referred to as the “non-phloem” phase of probing. EPG parameters describing aphid probing behavior were calculated manually and individually for every aphid.

#### 4.2.6. Statistical Analysis

The data deriving from the choice-test for freely moving aphids (aphid settling deterrent activity) were analyzed using Student’s *t*-test. If aphids showed clear preference for the leaf treated with the tested compound (*p* < 0.05), the compound was described as having attractant properties. If aphids settled mainly on the control half of the leaf (*p* < 0.05), the compound tested in the respective choice-test was stated a deterrent. The relative index of deterrence (DI) was calculated according to the formula DI = C − T/C + T, where C is the number of aphids that remained on control half of the leaf, and T is the number of aphids that remained on the treated half of the leaf. The value of DI ranged between 1 (ideal deterrent) and −1 (ideal attractant) [60].

The data deriving from the no-choice test for freely moving aphids (aphid repellent activity) were analyzed using one-way ANOVA followed by the Tukey test.

Data deriving from the no-choice EPG test for tethered aphids (probing and feeding deterrent activity) were analyzed by the Kruskal–Wallis test and post-hoc multiple comparisons of mean ranks for all groups (Dunn’s test) due to failure to meet the assumptions of analysis of variance. The Kruskal–Wallis test is a non-parametric alternative to the one-factor ANOVA test for independent measures, and it is commonly used to analyze data deriving from EPG recordings of aphid probing [67]. Although the mean and SE values given in Table 1, Table 2 and Table 3 represent non-Gaussian data, the statistical analysis was performed using the non-parametric test in which all individual data were included.

All statistical calculations were performed using StatSoft, Inc. (2014) STATISTICA (data analysis software system, version 12) (Tulsa, OK, USA).

## Figures and Tables

**Table 1:** Effect of citral and citral-derived lactones on the settling behavior of *Myzus persicae*. Aphids had free access to treated and untreated half-leaf sections for 24 h. Numbers represent mean (±SE) number of aphids that settled on treated (T) and untreated control (C) halves of the leaves at 15 and 30 min and 1, 2, and 24 h after aphids had access to the leaves; *n* = 8 (20 aphids per replicate); *p* < 0.05 denotes statistically significant differences (Student *t*-test).

Compounds		Number of Aphids				
		15 min	30 min	1 h	2 h	24 h
(**1**) citral	treated	2.1 (±0.3)	2.9 (±0.4)	3.1 (±0.4)	2.3 (±0.3)	1.4 (±0.4)
	control	10.8 (±1.0)	11.0 (±1.1)	9.8 (±1.0)	10.9 (±0.3)	8.0 (±1.3)
	*p*	0.0000	0.0000	0.0000	0.0001	0.0002
(**4**) β,γ-dimethyllactone	treated	3.9 (±0.8)	4.5 (±0.6)	5.0 (±0.6)	5.0 (±0.8)	3.9 (±0.7)
	control	11.0 (±1.0)	11.9 (±1.2)	11.3 (±1.1)	11.9 (±1.0)	8.0 (±1.1)
	*p*	0.0001	0.0001	0.0002	0.0001	0.0064
(**5**) α-methylenelactone	treated	1.8 (±0.5)	1.5 (±0.4)	1.0 (±0.4)	1.6 (±0.6)	0.6 (±0.3)
	control	11.0 (±1.0)	11.8 (±1.1)	11.3 (±1.1)	9.5 (±1.2.)	5.0 (±1.7)
	*p*	0.0000	0.0000	0.0000	0.0000	0.0214
(**6**) γ-methylenelactone	treated	6.6 (±1.0)	5.8 (±1.1)	6.6 (±1.1)	6.4 (±0.8)	3.3 (±0.6)
	control	7.9 (±1.2)	7.8 (±1.1)	9.1 (±1.2)	9.1 (±1.0)	5.1 (±1.0)
	*p*	0.4371	0.2343	0.1448	0.0486	0.1070
(**7**) α,γ-dimethylenelactone	treated	3.5 (±0.7)	4.5 (±0.7)	5.1 (±0.8)	3.9 (±0.7)	1.8 (±0.5)
	control	8.0 (±0.6)	9.0 (±0.7)	8.4 (±0.9)	9.0 (±0.5)	4.4 (±0.7)
	*p*	0.0002	0.0003	0.0170	0.0001	0.0086

**Table 2:** Responses of *Myzus persicae* to citral and citral-derived lactones during the 15 min video recorded free access to treated and untreated (control) leaves. Values represent means (±SE), *n* = 16; different letters in rows show significant differences at *p* < 0.05 (Kruskal–Wallis test).

Aphid Behavior	Control	(1) Citral	(4) β,γ-Dimethyllactone	(5) α-Methylenelactone	(7) α,γ-Dimethylenelactone
Total time on the leaf (min)	14.6 (±0.2) a	5.2 (±1.3) b	13.9 (±0.5) a	11.4 (±1.2) ab	13.3 (±1.1) a
Total probing time (min)	9.2 (±0.8) a	3.0 (±1.2) b	8.8 (±0.9) a	6.6 (±4.5) ab	10.0 (±1.0) a
Probe duration (min)	2.1 (±0.4) a	0.7 (±0.2) b	1.8 (±0.3) ab	1.6 (±0.4) ab	3.8 (±0.7) a
Number of probes (min)	5.6 (±0.6) a	3.8 (±0.9) ab	6.5 (±0.6) ac	5.3 (±0.7) abc	3.0 (±0.3) b
Time to the first probe (sec)	14.4 (±2.7) a	11.6 (±2.2) a	13.1 (±1.8) a	11.8 (±3.6) a	20.9 (±3.4) a

**Table 3:** Probing behavior of *Myzus persicae* on citral and citral-derived lactone-treated leaves (means ± SE). The mean and SE values are a representation of non-Gaussian data, but the statistical analysis was done by non-parametric tests in which all individual data were included; *n* = number of replications; C = pathway in non-phloem tissues; E1 = phloem salivation; E2 = phloem sap ingestion; F = derailed stylet activities; G = xylem sap ingestion. Different small letters in rows show significant differences in the values of specific parameters among aphids on plants treated with individual citral derivatives (Kruskal–Wallis test, *p* < 0.05).

Aphid Probing Behavior	Control	(1) Citral	(4) β,γ-Dimethyllactone	(5) α-Methylenelactone	(7) α,γ-Dimethylenelactone
**General Aspects of Aphid Probing Behavior ^1^**					
Total duration of probing C + E1 + E2 + F + G (h)	7.4 (±0.2) a *n* =16	4.2 (±0.4) b *n* = 16	7.1 (±0.3) a *n* = 16	4.1 (±0.5) bc *n* = 16	6.5 (±0.3) ac *n* = 16
Total duration of pathway phase C + F (h)	2.6 (±0.5) *n* = 16	2.9 (±0.3) *n* = 16	2.5 (±0.6) *n* = 16	3.3 (±0.4) *n* = 16	3.4 (±0.4) *n* = 16
Total duration of phloem phase E1 + E2 (h)	4.5 (±0.7) a *n* = 16	1.3 (±0.3) b *n* = 16	4.6 (±0.7) ab *n* = 16	0.5 (±0.4) bc *n* = 16	3.0 (±0.4) ab *n* = 16
Total duration of sap ingestion phase E2 (h)	4.5 (±0.7) a *n* = 16	1.2 (±0.3) b *n* = 16	4.6 (±0.7) ab *n* = 16	0.5 (±0.1) bc *n* = 16	2.8 (±0.4) ab *n* = 16
Total duration of xylem phase G (min)	11.4 (±6.0) *n* = 16	3.7 (±3.6) *n* = 16	4.8 (±3.3) *n* = 16	13.3 (±13.3) *n* = 16	8.2 (±3.3) *n* = 16
Phloem phase index (E1 + E2)/(C + E + G)	0.62 (±0.09) a *n* = 16	0.31 (±0.06) ab *n* = 16	0.64 (±0.09) a *n* = 16	0.13 (±0.05) b *n* = 16	0.46 (±0.06) a *n* = 16
Phloem salivation index E1/(E1 + E2)	0.01 (±0.00) a *n* = 16	0.08 (±0.02) ab *n* = 16	0.01 (±0.00) ac *n* = 16	0.19 (±0.09) b *n* = 16	0.05 (±0.03) ab *n* = 16
Number of probes (#)	11.5 (±2.7) a *n* = 16	38.7 (±3.1) b *n* = 16	12.4 (±3.4) a *n* = 16	37.2 (±5.0) b *n* = 16	23.2 (±3.2) ab *n* = 16
**Aphid Probing Behavior before the First Phloem Phase**					
Time from start of EPG to the first probe (min)	1.3 (±0.4) *n* = 16	12.4 (±6.0) *n* = 16	2.9 (±2.1) *n* = 16	25.6 (±13.3) *n* = 16	1.3 (±0.6) *n* = 16
Duration of first probe (min)	103.1 (±47.3) *n* = 16	14.6 (±5.9) *n* = 16	77.5 (±40.6) *n* = 16	8.0 (±3.3) *n* = 16	14.7 (±7.0) *n* = 16
Time from first probe to first phloem phase ^2^ (h)	1.5 (±0.4) a *n* = 15	3.8 (±0.6) b *n* = 13	1.1 (±0.4) a *n* = 13	2.5 (±0.8) ab *n* = 7	2.0 (±0.4) ab *n* = 16
Total duration of no probing after first probe ^2^ (min)	14.7 (±5.6) a *n* = 15	123.2 (±27.8) b *n* = 13	15.6 (±6.0) a *n* = 13	66.6 (±41.7) ab *n* = 7	51.5 (±15.5) ab *n* = 16
Number of probes ^2^	5.2 (±1.7) a *n* = 15	24.3 (±4.3) b *n* = 13	5.2 (±2.5) a *n* = 13	12.9 (±4.7) ac *n* = 7	9.9 (±0.3) ab *n* = 16
Number of probes ^2^ < 2 min	1.8 (±0.6) a *n* = 15	14.3 (±3.1) b *n* = 13	2.7 (±1.5) a *n* = 13	7.4 (±4.5) ab *n* = 7	4.8 (±1.5) ab *n* = 16
Number of probes ^2^ 2–10 min	2.1 (±0.7) a *n* = 15	7.9 (±1.6) b *n* = 13	2.3 (±1.1) a *n* = 13	4.3 (±1.8) ab *n* = 7	3.8 (±1.0) ab *n* = 16
Number of probes ^2^ > 10 min	1.3 (±0.4) a *n* = 15	2.1 (±0.8) a *n* = 13	0.4 (±0.2) a *n* = 13	1.2 (±0.4) a *n* = 7	1.3 (±0.3) a *n* = 16
**Aphid Probing Behavior Associated with Phloem Phase**					
Duration of first phloem phase E1 or E1+E2 ^2^ (h)	2.8 (±0.9) a *n* = 15	1.0 (±0.3) a *n* = 13	3.7 (±0.9) a *n* = 13	0.2 (±0.1) b *n* = 7	0.6 (±0.3) a *n* = 16
Duration of first phloem ingestion phase E2 ^3^ (h)	2.8 (±0.9) a *n* = 15	1.1 (±0.3) a *n* = 12	4.0 (±0.8) ab *n* = 13	1.3 (±1.1) a *n* = 5	0.4 (±0.2) ac *n* = 16
Duration of first sustained phloem sap ingestion phase E2 > 10 min ^4^ (h)	3.8 (±0.9) a *n* = 14	1.6 (±0.3) b *n* = 10	4.5 (±0.8) a *n* = 13	1.4 (±1.1) b *n* = 5	1.5 (±04) ab *n* = 15

^1^ All replications were included in analysis; ^2^ only replications containing at least one phloem phase E1 were included in analysis; ^3^ only replications containing phloem phase E2 were included in analysis; ^4^ only replications containing phloem phase E2 >10 min were included in analysis.

## Supplementary Materials

The following are available online at https://www.mdpi.com/1422-0067/21/21/8029/s1.
