# Supplementary material for: Repellent and Antifeedant Activities of Citral-Derived Lactones against the Peach Potato Aphid
Source: Int J Mol Sci. 2020 Oct 28;21(21):8029. doi: 10.3390/ijms21218029 (PMC7663017; doi:10.3390/ijms21218029)
Supplement: Supplementary file 1 [file ijms-21-08029-s001.pdf]

Supplementary Material

Repellent and Antifeedant Activities of Citral-Derived Lactones against the Peach Potato Aphid

Chemical Syntheses

trans-5-(iodomethyl)-4-methyl-4-(4-methylpent-3-en-1-yl)dihydrofuran-2(3H)-one (3)

To a solution of unsaturated acid (**2**, 10 g, 0.051 M) in 100 mL of diethyl ether, 100 mL of saturated solution of sodium bicarbonate was added, and with intensive stirring at room temperature an aqueous solution of iodine 25.4 g (0.1 M) in potassium iodide 16.6 g (0.1 M) in 100 mL of water was dropped. The dropping was carried out for four hours until the ether layer was permanently iodine stained. Then, 10 g (0.16 M) of sodium thiosulphate was added to the solution, stirring intensively for next 10 min. Then the organic layer was separated and washed with water and brine. After drying over Na_2SO_4 and evaporation of the solvent, 14.5 g (0.045 M) of mixture (*cis* 15% and *trans* 85%) iodolactones was obtained. The raw product was subjected to column chromatography (silica gel, hexane:diethyl ether, 2:1). The pure isomer *trans* (11.1 g, 0.034 M, yield 66%) was obtained. Physical and spectral data of product (**3**) are given below:

n_D^{20}: 1.5188; ^{1}H NMR ($CDCl_3$): 5.01 (1H, t, J = 7,0 Hz; –CH_2–CH_2–C<u>H</u>=); 4.37 (1H, m, –C<u>H</u>(O)CH_2I); 3.14–3.34 (2H, m; –CH(O)C<u>H</u>$_2$I); 2.40 i 2.32 (2H, two d, *J* = 17.2 Hz; –CH_2C(O)O– AB system); 1.91–2.01 (4H, m, –C<u>H</u>$_2$–C<u>H</u>$_2$–CH=); 1.59 and 1.56 (two s, 6H, –CH=C(CH_3)$_2$); 1.02 (3H, s, –CH_3–4); IR (film) cm-1: 1785 (s), 1220 (w); 1173 (m); 979 (m); MS (m/z) (%): 323 [M+H] (32); 195 (100); 177 (27); 139 (28); 135 (89); 121 (18); 107 (21); 81 (28).

trans-4,5-Dimethyl-4-(4-methylpent-3-enyl)dihydrofuran-2(3H)-one (4)

To a solution of *trans*-iodolactone (**3**, 4.0 g, 0.015 M) in 50 mL of anhydrous benzene, 4.7 mL (0.020 M) of tri-*n*-butyl tin hydride and a catalytic amount of azaizobutylonitrile were added. The reaction mixture was refluxed within 10 h. Then the solvent was evaporated, and the product was purified by column chromatography (silica gel, hexane–diethyl ether, first 30:1 and finally 2:1). Pure product (1.76 g, 0.009 M, yield 60%) was obtained. Physical and spectral data of product (**4**) are given below:

n_D^{20}: 1.4528; ^{1}H NMR (C_6D_6): 5.03 (1H, t, *J* = 5.0 Hz; –CH_2–CH_2–C<u>H</u>=); 3.77 (1H, q, *J* = 6,5 Hz; –C<u>H</u>(CH_3)O–); 1.96 (2H, s, –CH_2C(O)O–); 1.73 (3H, s, one of (CH_3)$_2$C=); 1.55 (3H, s, one of (CH_3)$_2$C=); 1.62–1.82 (2H, m, –CH_2–C<u>H</u>$_2$–CH=); 0.98 (1H, m, *J* = 13.4; one of –C<u>H</u>$_2$–CH_2–CH=); 0.94 (3H, d, *J* = 6.5 Hz; –CH(CH_3)O–); 0.79 (1H, m, *J* = 13.4 Hz; one of –C<u>H</u>$_2$–CH_2–CH=); 0.60 (3H, s, CH_3–4); IR (film) cm-1: 1800 (s); 1395 (m); 1200 (m); 1080 (m); MS (m/z) (%): 197 [M+H] (17); 179 (6); 163 (3); 153 (19); 137 (100); 121 (23); 109 (16); 95 (21); 81 (28); 69 (45); 55 (14); 41 (46).

4,5-dimethyl-3-methylene-4-(4-methylpent-3-en-1-yl)dihydrofuran-2(3H)-one (5)

Lactone (**4**, 0.5 g, 0.0025 M) was placed in round-bottom flask and dried with nitrogen. Then a tenfold excess (0.025 M, 12.5 mL) of 2M solution of methyl methoxymagnesium carbonate (MMC) in DMF was added. The reaction mixture was heated under nitrogen atmosphere at the solvent boiling temperature for 48 h. The reaction progress was monitored by TLC. After that time, the reaction mixture was poured into a beaker containing 50 mL of ether and 50 mL of 2M hydrochloric acid with intensive stirring. The organic layer was separated and the water layer was additionally extracted with diethyl ether (2 × 50 mL). The combined ethereal solutions were washed with water and brine to a neutral pH and dried over anhydrous Na_2SO_4. After evaporation of the solvent, 0.5 g of diastereoisomeric mixture of α-carboxylactones was obtained. At the same time, a methylation mixture was prepared, consisting of freshly distilled N-methylaniline (3 mL), formalin (6 mL), acetic acid (8 mL), and sodium acetate (0.2 g), which was also used in both methylation reactions. The α-

carboxylactone mixture was then dissolved in 5 mL of methylenation mixture and stirred at 40 °C for eight hours. The reaction progress was monitored by TLC as well as by carbon dioxide bubbles releasing in the mixture as a product of decarboxylation reaction. After 8 h, to the reaction mixture 80 mL of ether was added. The organic solution was washed with 3 × 50 mL of 2M HCl sodium bicarbonate solution, water, and brine leading to neutral pH. After drying over Na_2SO_4 and evaporation of the solvent, the product was purified by column chromatography (hexane:diethyl ether from 10:1 to 4:1). In this way, α-methylenelactone **5** (0.32, 0.0015 M, 63% yield) was obtained. Physical and spectral data of product (**5**) are given below:

n_D^{20} : 1.4757; ^{1}H NMR (C_6D_6) δ: 6.23 (1H, s, one of >$C{=}CH_2$); 5.01 (1H, t, *J*=6.9 Hz; $(CH_3)_2C{=}C\underline{H}$-); 5.00 (1H, s, one of >$C{=}CH_2$); 3.98 (1H, q, *J*=6.5 Hz; (CH_3)-$C\underline{H}O$-); 1.68-1.84 (2H, m; -CH_2-$C\underline{H}_2$-CH=); 1.70 (3H, s; one of $(CH_3)_2$-C=CH-); 1.52 (3H, s; one of $(CH_3)_2C{=}CH$-); 1.19-1.28 (2H, m, -CH_2-CH_2-CH=); 0.89 (3H, d, *J*=6.5 Hz; -(CH_3)-CHO-); 0.69 (3H, s, CH_3-4); IR (film, cm-1): 1776 (s), 1688 (m), 1604 (s), 1232 (m), 1172 (m). MS (m/z) (%): 209 [M+H] (100); 191 (19); 181 (3); 165 (60); 137 (13); 124 (23); 109 (23); 95 (8); 81 (6); 63 (5); 43 (10).

4-methyl-5-methylene-4-(4-methylpent-3-en-1-yl)dihydrofuran-2(3H)-one (6)

To a solution of *trans*-iodolactone (**3,** 4 g, 0.015 M) in 50 mL methylene chloride, 3.3 mL (0.02 M) of DBU was added and stirred at room temperature for 2 h. When the reaction was completed (TLC), 50 mL of 2 M hydrochloric acid solution was added to the reaction mixture. The methylene chloride solution was separated from the product, and the water solution was extracted additionally with 2 × 50 mL of methylene chloride. The combined organic layers were washed with sodium bicarbonate, water, and brine. After drying over sodium sulfate and evaporation of the solvent, the product was evaporated, and crude product was purified by column chromatography (silica gel, hexane:diethyl ether from 10:1 to 2:1). Pure product (**6**) (2.2 g 0.0115 M, 77% yield) was obtained. Physical and spectral data of product (**6**) are given below:

n_D^{20} : 1.4790; ^{1}H NMR ($CDCl_3$): 5.05 (1H, t, *J*=6.9 Hz -$CH{=}C(CH_3)_2$; 4.72 (1H, d, *J*=2.6, one of >$C{=}CH_2$); 4.25 (1H, d, *J*=2.6 Hz one of >$C{=}CH_2$); 2.57 i 2.42 (2H, two d, *J*=17.8 Hz –$CH_2C(O)$- AB system); 1.92-2.04 (2H, m, -CH_2-$C\underline{H}_2$-CH=); 1.67 i 1.58 (6H, two s, -$CH{=}C(CH_3)_2$); 1.50-1.57 (2H, m, -$C\underline{H}_2$-CH_2-CH=); 1.30 (3H, s, CH_3-); IR (film) cm-1: 1808 (s); 1667 (m); 1250 (w); 1170 (m); 1080 (m); MS (m/z) (%): 195 [M+H] (8); 177 (2); 151 (5); 135 (4); 119 (1); 112 (16); 95 (9); 82 (100); 67 (95); 55 (12); 41 (37)

4-methyl-3,5-dimethylene-4-(4-methylpent-3-en-1-yl)dihydrofuran-2(3H)-one (7):

α,γ–Dimethylenelactone (**7**, 0.33 g, 55%) was obtained from 0.5 g of lactone **6** in the same procedure as that described for lactone **5**. Physical and spectral data of product (**7**) are given below:

n_D^{20} :1.4757; ^{1}H MNR (C_6D_6): 6.06 (1H, d, *J*=2.7 Hz; one of $CH_2{=}C$< on C-3, 5.32 (1H, d, *J*=2,7 Hz; one of $CH_2{=}C$< on C-3), 4.98 (1H, t, *J*=7,0 Hz; -CH_2-CH_2-$C\underline{H}$=); 4.61 (1H, d, *J*=2,6 Hz; one of $CH_2{=}C$< on C-5); 4.20 (1H, d, *J*=2.6 Hz one of $CH_2{=}C$< on C-5); 1.84 (3H, s, one of $(CH_3)_2C$=); 1.77-1.81 (1H, m; one of -CH_2-$C\underline{H}_2$-CH=); 1.65 (3H, s; one of $(CH_3)_2C$=); 1.55-1.59 (1H, m; one of -CH_2-$C\underline{H}_2$-CH=); 1.50 (3H, s, CH_3-4); 1.26-1.33 (2H, m; -$C\underline{H}_2$-CH_2-CH=); IR (film, cm-1): 1776 (s); 1688 (m); 1604 (s); 1232 (m); 1172 (m).
